# Some improvements but a long way to go: a national survey of local authorities on the provision of social care for people released from prison

**DOI:** 10.1186/s40352-024-00304-6

**Published:** 2024-11-29

**Authors:** Claire Hargreaves, Amy Roberts, Wendy Taylor, Katrina Forsyth, Catherine Robinson, Jennifer Shaw, Susan Tucker

**Affiliations:** 1 Lancaster University, Lancaster, UK; 2 University of Manchester, Manchester, UK; 3 University of Chester, Chester, UK

**Keywords:** Prisoners, Social care, Release, Community, Survey

## Abstract

The provision of social care for people in prison in England has historically been lacking. Seeking to address this, the 2014 Care Act clarified that local authorities are responsible for identifying, assessing and meeting prisoners’ social care needs. Against this background, in 2020 we undertook a survey to explore the emerging services for this group. Eighty-six (57%) local authorities responded. A mixed methods approach was taken. Numerical data were analysed through descriptive statistics with comparisons made to the previous survey. An inductive approach to thematic analysis was used to analyse the free text responses. The findings revealed some improvements since the 2015/16 surveys, including the wider introduction of self-referral systems, the success of peer supporters in identifying people in need of social care and greater multi-disciplinary working. However, other issues remained stubbornly persistent, including a dearth of systematic processes to identify those needing social care on release from prison, a lack of timely information sharing and disputes over the sending and receiving authorities’ responsibilities. There were also particular concerns about the shortage of appropriate accommodation for people leaving prison. Perhaps the most striking finding, however, was how little most authorities knew about this population. Building on discussions in previous papers, we explore three ways in which arrangements could be strengthened for this group: the collection of better data, the wider use of release on temporary licence and the greater employment of technology in planning people’s release.

## Introduction

At more than 11.5 million people, the global prison population is bigger than ever and numbers are increasing in four of the five continents (Charles, 2015; Penal Reform International, 2022; Walmsley, 2018). England and Wales are no exception. In the last 30 years, the prison population has grown by 75% and estimates suggest that by March 2025 there will be around 95,000 people in custody (Ministry of Justice, 2023). The vast majority of prisoners will be released at some point (Ministry of Justice, 2022). However, re-integrating into the community can be fraught with difficulty. Numerous studies highlight the multiple challenges people face on release from prison, including securing stable housing, obtaining employment, accessing health services and reconnecting with family and friends (Ahmad & Eves, 2020; Hyde et al., 2022; Hu et al., 2020). It is thus not surprising that they have a high risk of adverse health and social outcomes at this time (Ahmad & Eves, 2020; Binswanger et al., 2012; Williamson, 2006; Zlodre & Fazel, 2012).

Social care services run by local authorities (LAs) (units of local government) can play a vital part in supporting people on release from prison. However, attention has typically focused on their role with housing, finance, benefits and families (Bradley, 2009; Agency et al., 2013). In contrast, very little attention has been given to the personal and practical social care support that people require to lead independent, fulfilling lives in the community. This is despite the fact that, as a consequence of demographic ageing, longer sentences and a surge of retrospective prosecutions for non-recent sex offences, the proportion of older people in prison is increasing, and with it the level of social care need (Her Majesty’s Inspectorate of Prisons & Care Quality Commission, 2018; Lee et al., 2019; Penal Reform International, 2022; Prais & Sheahan, 2019). Older people in prison (typically defined as 50+) are considerably more likely than younger prisoners to experience long-term illness, sensory impairment and disability (Hayes et al., 2013; House of Commons Justice Committee, 2020). In one study of 482 male prisoners in Lancashire, a fifth of older prisoners (compared with a tenth of younger prisoners) reported difficulty maintaining their personal hygiene, dressing and/or getting around the prison safely, whilst a significant minority lacked meaningful occupation and/or had problems forming/maintaining relationships (Tucker et al., 2019). Moreover, the proportion experiencing problems on release is likely to be still higher as many people conceal their difficulties in prison for fear of appearing vulnerable, whilst others who just about manage within the structured prison regime will need social care in the community (Anderson & Cairns, 2011; Cornish et al., 2016; Her Majesty’s Inspectorate of Prisons & Care Quality Commission, 2018). Older people are not the only subgroup who may need social care on release, however; younger adults with neurodivergent conditions, physical disabilities or mental health problems can also require social care (Local Government Association & National Offender Management Service, 2014; Skills for Care, 2015).

Despite such growing need, a number of reports at the start of this century highlighted a marked want of social care for people in prison in England (Anderson & Cairns, 2011; Her Majesty’s Inspectorate of Prisons, 2004, 2008, 2009; Parker, McArthur & Poxton, 2007). One survey of prison governors, for instance, found local authority social care staff were involved in assessing and meeting people’s social care needs in just a quarter of prisons, whilst involvement in people’s release was still rarer (Local Government Association & National Offender Management Service, 2014). Although some prisons appreciated the benefits of social care, many lacked care levels taken for granted in the community, leaving people reliant on help from other prisoners despite the absence of training or supervision (Pitt, 2011).

**Fig. 1:**
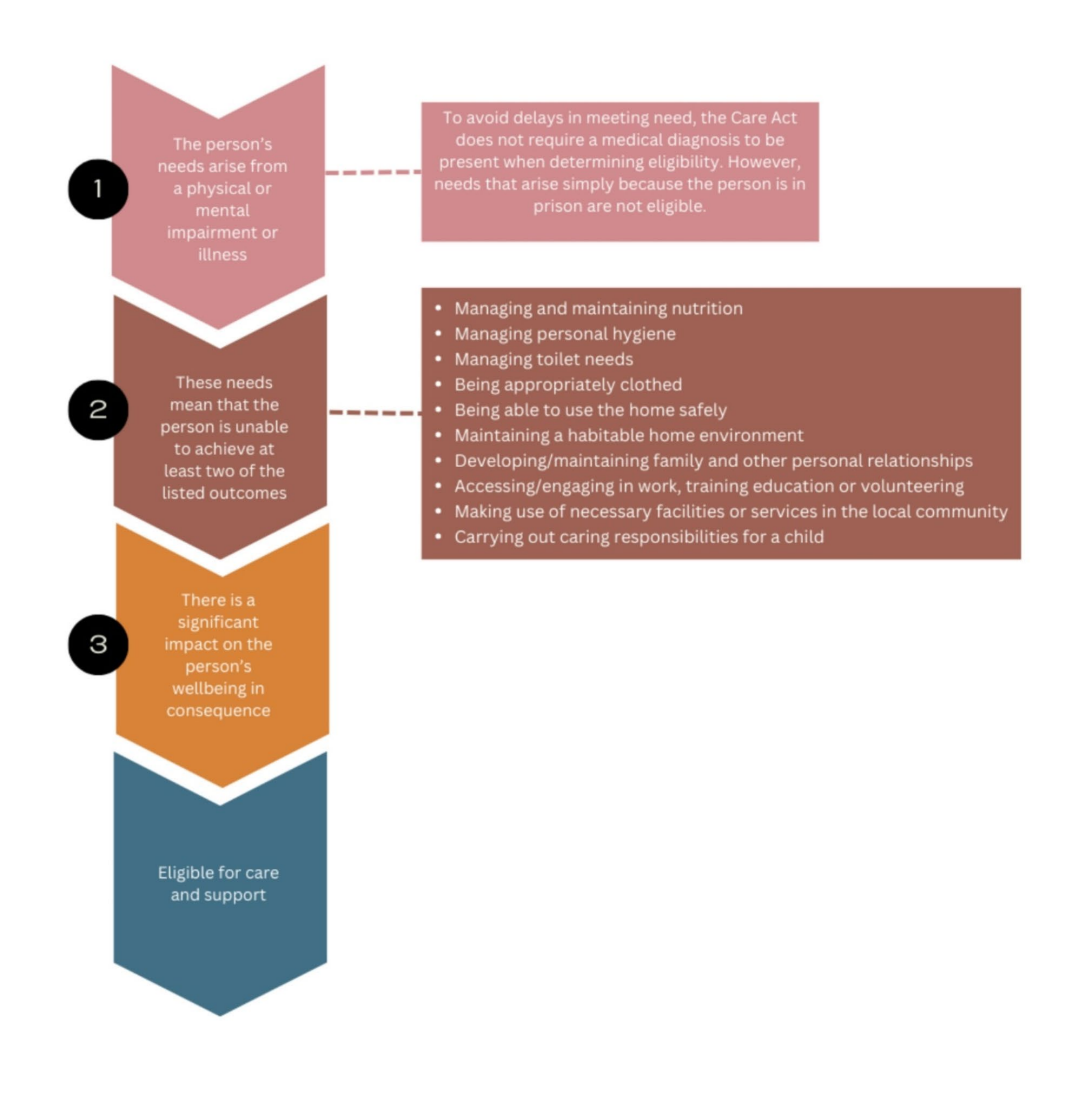
The care and support eligibility criteria

In the past, this situation was generally attributed to uncertainty about whether or which local authorities were responsible for providing social care in prisons (Department of Health, 2014; Lee et al., 2019; Pettus-Davis, 2012). However, the 2014 Care Act made it clear that local authorities with prisons in their catchment area have a duty to provide social care and support for people in custody who meet the national eligibility criteria (see Fig. 1). The Care Act 2014 suggests a social care need is present when the individual is unable to achieve two or more of the acts defined outcomes (listed in step two of Fig. 1) due to a physical or mental impairment or illness, and there is significant impact on the person’s wellbeing in consequence. Further, all local authorities are responsible for the social care of people who move into their area with a package of care on release from prison, with the ‘sending’ authority expected to liaise with the ‘receiving’ authority when people are released into different geographical areas. The receiving authority should then undertake a community care assessment and make the necessary arrangements (or, if they are unable to do this before the person is released, continue to provide the care suggested by the sender until such time as a community assessment is carried out). The Act did not, however, specify the method of service delivery, and the number of people requiring social care was unclear, there no national data collection.

In 2015/16, two national surveys of the early arrangements local authorities had put in place to meet these new responsibilities suggested that the provision of social care for people in custody *had* improved. Most authorities had processes in place for identifying prisoners with social care needs, specialist social care staff were undertaking the majority of assessments and around 600–800 people per year were receiving a commissioned care package in custody, albeit this was thought to be just the tip of the iceberg. Moreover, arrangements for people with social care needs released from prison appeared far less developed, with a significant minority of respondents identifying difficulties with case finding, liaising with other authorities and coordinating care with other agencies. Indeed, it was not clear that all authorities understood their new responsibilities (Tucker et al., 2018; Robinson et al., 2021) and a subsequent thematic report (Her Majesty’s Inspectorate of Prisons & Care Quality Commission, 2018) made a series of suggestions to improve provision. These included prisons and local authorities implementing prompt, ongoing and effective systems for identifying prisoners’ social care needs *throughout* their stay in prison, and robust discharge processes, encompassing effective information sharing and links to appropriate support services. An international scoping review of the support provided for people with social care needs on release from prison, however, exposed the extremely limited evidence on which practice for this group was still based. Indeed, the review concluded that processes of care were typically poorly understood, limited information was available on the implementation and experience of specific initiatives, and data on prevalence and outcomes were lacking (Tucker et al., 2024).

Against this background, this paper reports the findings of a subsequent local authority survey conducted some five years after the implementation of the Care Act with the aim of establishing the systems, processes and structures in place to recognise, plan for, and support the release of prisoners with social care needs following its implementation and enabling local authorities to learn from others’ successes and challenges. Whilst the data (and the Act) relate to England alone, the problems the Act addresses are anticipated to have a resonance for countries worldwide.

## Method

The research formed part of a larger study of the social care needs of people released from prison commissioned by the National Institute for Health Research (NIHR) School for Social Care Research (C969/CM/UMCR-P136). There were three main stands: the reported survey; the aforementioned scoping review; and qualitative interviews with key professionals in four good practice sites. This paper reports the findings of the local authority survey which aimed to establish the systems, processes and arrangements in place to identify, plan and support the release of prisoners with social care needs.

### Survey recruitment

An introductory letter and Word questionnaire were emailed to the Director of Adult Social Services in each local authority in England in early 2020 (*n* = 151), asking that they forward these to the person in their organisation with the most local knowledge about the provision of social care for people released from prison. Data collection was paused in the spring and summer of that year because of Covid, but resumed in the autumn. Data collection closed at the end of the year. The study was supported by the Association of Directors of Adult Social Services (ADASS) and promoted in their Bulletin.

### Content

Separate questionnaires were devised for local authorities with and without prisons in their catchment area. These were informed by and built on the earlier local authority surveys (Robinson et al., 2021; Tucker et al., 2018) and literature review (Tucker et al., 2024) and were piloted with and revised in accordance with the comments of six local authorities prior to final dissemination. Befitting an area about which little was known, they had a semi-structured format containing a mix of (mostly) open-ended and pre-coded items and focused on the authorities’ practice in the preceding 12 months. The questionnaire for authorities containing prisons contained sections on identifying people with social care needs in prison, the prevalence and situation of people with social care needs on release (information not collected in previous surveys) and arrangements for their release; all authorities were asked about the number of and arrangements for people with social care needs released into their community. Where authorities had multiple prisons in their catchment area and practice differed between them, respondents were asked to state what happened in each establishment.

### Analysis

The responses were inputted into a bespoke Excel database from the completed survey documents. Double data entry checks were performed, with an error rate of 0.7%. Numerical and pre-coded data were analysed with descriptive statistics using SPSS for Windows version 27 and where the data allowed, the results were compared with the findings from the previous surveys (Robinson et al., 2021; Tucker et al., 2018). An inductive approach was used to analyse the free text responses, allowing the researchers to organise the data without losing sight of the detail (Braun & Clarke, 2006, 2012). This was an iterative process whereby four team members (ST, WT, KF, AR) familiarised themselves with the data by reading and re-reading the responses, identifying the main themes and illustrative examples.

## Findings

### Sample characteristics

Eighty six local authorities returned completed questionnaires, a 57% response rate. These comprised 40/59 (68%) authorities with prisons in their catchment area and 46/92 (50%) without. Of those authorities containing prisons, 23 contained one prison, eight contained two and nine had three or more. Respondent authorities served prisons housing male prisoners in all four security categories as well as prisons for women.

The role of the respondent varied, with the majority holding the head of department role (e.g. head of adult social care, head of social work, and head of complex care) or service manager role (e.g. service manage for adult social care, care management access, and service manager assessment and hospital discharge). Further roles, amongst others, included, director of adults’ services, assistant director, care act implementation lead, chief officer and health and wellbeing development officer.

### Identifying individuals with social care needs pre-release

#### Identifying individuals with social care needs on entry to prison

Processes for identifying people with social care needs on entry to prison largely mirrored those employed in 2015/16, with the initial screening process undertaken by health and/or prison staff. No authority reported the involvement of specialist social care staff at this stage and four respondents explicitly stated that they had not had any involvement in determining the questions asked. However, 29/39 (74%) authorities that answered a specific question about the ability of people in custody to self-refer had a process for this, suggesting this was becoming more widespread. (Whilst information on self-referrals was not specifically sought in earlier surveys, less than a third of previous respondents had mentioned these.) One authority also employed an adult social care peer advisor in the first night unit to identify people who required social care (Authority ID 923768).

The proportion of social care referrals estimated to come from the reception process varied greatly. Of 28 authorities that provided this information, nine (32%) had received no referrals from reception, whilst a further nine (32%) had received less than 20 per cent. In contrast, six authorities (21%) said at least 50 per cent of referrals came from this source. Eight authorities could not answer this question and a further four did not respond.

#### Identifying individuals with social care needs during their prison stay

Formal processes for identifying people who developed social care needs during the course of their prison stay remained lacking. Whilst many respondents provided a long list of professionals who could refer to the local authority (predominantly health, prison and probation staff), there was little if any indication of systematic case finding further to reception and no mention of screening pre-release. This was despite the fact that more than half of respondents (*n* = 24) said there were particular subgroups of people who, whilst not receiving social care in prison, were likely to do so on release. This included people with significant mental health needs, learning disabilities, dementia and autism who have been in prison for long periods of time.

Several authorities highlighted their attendance at multi-disciplinary/complex case meetings as another source of referrals and wrote positively about the relationships they had developed with other staff. However, many of these meetings appeared to focus on people at high risk to others (e.g. people subject to Multi-Agency Public Protection Arrangements (MAPPA)), as opposed to people at high risk themselves. The involvement of trained peer supporters did, however, appear to be helpful in identifying people in need of social care and support on release, with such individuals said to:

…. have an understanding of the needs of individuals who may require support, and the referral process in that particular prison (Authority ID 950401).

Further, in one authority social care practitioners held monthly open drop-in sessions for prisoners to meet with social care practitioners and discuss concerns, noting that:

…. using an open forum ensures regular access to our services for advice and guidance as well as formal assessments (Authority ID 966278).

The number of people with social care needs on release from prison thirteen authorities (32% of the responding 40 authorities with prisons) reported collecting data on the number of prisoners with eligible social care needs released to the community, whilst a further six respondents did not know if their authority collected such data. The former 13 authorities had released 60 prisoners with social care needs in the previous 12 months (minimum zero, maximum 15). A further 20 of the responding 40 authorities (with prisons) provided estimates of the number of prisoners with eligible social care needs released in the past year, suggesting a further 146 individuals were released with eligible social care needs (minimum zero, maximum 25). Estimates of the proportion of individuals with eligible social care needs released into communities served by other authorities were high, with 20 of the responding 40 authorities (with prisons) saying this applied to half or more releases and five to all. Thirteen local authorities could not provide an estimate however, whilst two did not answer the question. Large numbers of authorities were also unable/failed to answer further questions on the percentage of people with eligible social care needs released into the community who had been homeless prior to prison (*n* = 28) or who moved into approved premises on release (*n* = 17).

### Release planning for social care needs

Thirty three authorities (82.5% of the responding 40 authorities with prisons) responded to a question asking how much notice (on average) they received that someone with eligible social care needs was due to be released to the community, with their responses suggesting this varied hugely depending on the individual and the type of prison. Where prisoners were subject to MAPPA arrangements, respondents typically received at least two months’ notice, but where people were on remand, they could be released with no notice at all. Indeed, the general impression was that authorities often lacked sufficient notice to make adequate plans:

In planning release Probation Officers may contact us days before the release making referrals…. In many cases we are not given sufficient notice to plan (Authority ID 488680).

Very little notice. We often find out after they have been released (Authority ID 521271).

When asked if certain subgroups were particularly difficult to plan for (and, if so, which) four authorities said ‘no’ and a further four did not answer the question. The remainder, however, highlighted a range of factors that made planning someone’s release especially challenging, either in terms of securing suitable accommodation or engaging them with services. These included the nature of the person’s crime (e.g. sex offences, arson), health status (people with learning disabilities, mental health problems, dementia, learning difficulties, autism), social situation (people with no fixed address, non-UK residents) and length of time in prison (short sentence prisoners, long-stay prisoners), with many prisoners having multiple and overlapping needs.

The use of multi-disciplinary planning for people with eligible social care was nevertheless variable, with 10 (25%) authorities saying this was very common, 15 (37.5%) fairly common and 15 (37.5%) not very common. Specific initiatives to prepare people with social care needs for release also appeared rare. Indeed, just three respondents reported such initiatives, with one providing sessions on how the social care system works (Authority ID 482357), another referring to the work of Recoop, a charity which aims to promote the care, resettlement, rehabilitation and mutual aid of older people in the criminal justice system (Authority ID 749040) and a third offering reablement sessions covering various aspects of daily life including cooking, exercise, dealing with loneliness and scam awareness (Authority ID 271275). The use of Release on Temporary Licence (ROTL), which enables people coming towards the end of their sentences to prepare for life beyond prison, also appeared limited, with just five (12.5%) authorities saying this was often/sometimes (as opposed to rarely/never) used. However, the experiences described by the former suggested it could be very helpful, albeit no authority made reference to the collection of any outcome data in response to this (or, indeed, any other) question:

Our Occupational Therapist has used ROTLs as an opportunity to assess an individual’s ability to manage in the community with essential tasks including road safety and managing money. This has been used as a graded assessment tool to assess needs and plan future services (Authority ID 966278).

…. it has been an opportunity assess to their abilities to manage in a different environment and moving forward provide future placements with full and open assessment of the risks, and how we would manage them (Authority ID 198275).

Mirroring findings from 2015/16, a significant minority of authorities reported difficulties transferring information about people with social care needs to other local authorities. Indeed, 15 of 38 (39%) authorities that answered this question said current measures were not working very/at all well, compared with nine out of 37 (24%) previously (Robinson et al., 2021). Authorities’ experiences again varied between individuals and organisations. However, several reported problems persuading other authorities (particularly those without prisons in their area) to accept referrals/undertake assessments or to plan in a timely manner and seven of the 35 authorities (20%) that answered a specific question about establishing ordinary residence said difficulties with this were very (as opposed to fairly or not) common, particularly where people were subject to Sect. 117 of the Mental Health Act (entitling a person to free aftercare further to discharge from hospital), had been in prison for a long time or were of no fixed address:

The main issue we experience are arguments about Ordinary Residence and the receiving local authority refusing to accept the referral. LAs will state that the authority where the prison is situated, are now responsible, particularly if the individual has been in custody for a long time… (Authority ID 923768).

The need for the person to be located within the receiving authority before the local authority will assess their needs can cause problems, as generally social workers don’t travel to conduct an out of county assessment (Authority ID 641117).

### Arrangements for people released from prison with social care needs

Ten of the 86 (12%) respondents in total (seven containing prisons, three without) said they collected data on the number of people with eligible social care needs released from a prison in another authority into their community, whilst 56 (65%) said they didn’t collect such data and 20 (23%) didn’t know. In combination, the former 10 authorities said they had received 70 such individuals in the previous 12 months (minimum zero, maximum 54), whilst estimates provided by 35 other authorities ranged from zero to fifty. In the vast majority of cases, referrals were made by the same route as other community referrals i.e. to initial contact/access teams. However, a handful of respondents noted that where MAPPA applied, referrals were often made to staff attending MAPPA meetings. Overall, just six (7%) of respondents were confident that their authority was always notified when an individual with social care needs was released to their authority from a prison elsewhere, with 38 (44%) somewhat confident and 42 (49%) not at all confident.

Authorities described three main problems with the receipt of people with social care needs from prisons in other authorities, two of which mirrored the problems experienced by the sending authorities. The first related to the lack of timely notification, precluding the development of an effective care plan (a concern also voiced in 2015/16), with particular problems when the individual’s discharge address was not known at point of referral:

The main issue we have is lack of contact [from the discharging authority] before the person is released. This is usually done just before they are due to be released or in some cases the person is released and we have not been informed about it. It leaves for very little planning for the individual and usually results in emergency placements being sought which are at times unsuitable for the person going into them (Authority ID 946750).

Second, over and above this, many authorities said that they were not given enough information about the individual, that the provided information failed to reflect the person’s needs and/or that it was difficult for them to undertake pre-release assessments, suggesting a number of reasons for this:

Limited information on assessments due to the difficulty of assessing when someone is in an institution (Authority ID 141699).

Incongruous interpretation and application of Social Care assessments in prisons in other local authorities (Authority ID 950401).

…. poor access to custodial environment to enable assessments to take place (Authority ID 923768).

Third, several respondents reported a shortage of suitable accommodation for people with social care needs released from prison:

Housing needs are difficult to meet (Authority ID 341273).

…. dependent on the offence sometimes no accommodation can be found and the individual is provided with a bed and breakfast accommodation until further work can be done… (Authority ID 946750).

Lack of appropriate housing… limited specialist care settings to manage risk (Authority ID 989000).

Finally, when asked if they had any specific initiatives to support people with social care needs released from prison into the community, almost two-thirds (62%) of respondents said no/failed to answer the question, whilst many of those that replied in the affirmative described services for ex-prisoners generally, rather than people with social care needs specifically. One authority, however, described an enhanced care and support team which supported:

.… individuals who are at risk of entering the criminal justice system or are being released back to X who have a diagnosis of autism and / or learning disability (Authority ID 923768).

whilst another noted that:

A recently commissioned home care service has identified care providers who will provider care and support within the prison environment and would look to continuity in care if the person required personal care and support once released into the community (Authority ID 641117).

## Discussion

In her foreword to *Priorities for Adult Social Work Research* (James Lind Alliance, 2018) Lyn Romeo (Chief Social Worker, England) wrote that the development and use of research and evidence to help commissioners and providers understand what works best, support decision making and challenge ingrained thinking had been one of her main goals since taking up post in 2013. This publication, in identifying the issues that practitioners’, users’ and carers’ considered most important to research was seen as vital in aiding this, with the need for evidence on the impact of the Care Act identified as a top ten priority. Despite its’s potential implications for people in prison, however, there remains a dearth of systematic information on the Act’s effect on practice for this group, particularly upon release (Tucker et al., 2024). Against this background, this paper provides important insights into the provision of social care for people released from custody some five years after the Act’s introduction.

## Summary of key findings

### Identifying social care needs pre-release

Initial screening was largely conducted by health care or prison staff on reception. Options for self-referral and active case finding were not widespread. Multi-disciplinary team meetings were considered to be an important aspect of identifying those who need social care. However, they generally focussed on those at high risk of harm. Peer supporters were seen to be another helpful way of identifying people with social care needs.

### Systems and process for the release planning of people in prison with social care needs

Only 32% of local authorities collected data on the number of individuals who were eligible for social care on release from prison and there was often a lack of sufficient notice to make plans for release. The Care Act largely focusses on social care in prison rather than release. Social care planning for release is reliant on knowledge about accommodation which is often not known until release is imminent. There are no standardised screening procedures for release which would identify any potential social care needs. Specific initiatives to prepare people with social care needs for release appeared rare. Therefore, in spite of the Care Act timely and effective release planning for those with social care needs is still relatively rare.

Mirroring findings from 2015/16, a significant minority of authorities reported difficulties transferring information about people with social care needs to other local authorities. This is because the Care Act may be deemed open to interpretation; there is a lack of standard operating procedures for transferring information and funding is limited.

Whilst the findings point to improvements in some areas (including the wider introduction of self-referral systems, the success of peer supporters in identifying people in need of social care and more multi-disciplinary working, particularly for people subject to MAPPA), there was no consistent approach across the country. Moreover, many of the concerns raised in 2015/16 remained stubbornly persistent, with the survey revealing an absence of systematic processes to identify people who whilst not requiring commissioned social care and support in prison, would do so upon release, a lack of timeliness in information sharing and ongoing disputes over sending and receiving authorities’ responsibilities. It also raised particular concerns about the shortage of appropriate accommodation for people leaving prison. Perhaps the most striking finding, however, was how little information most authorities had about this population, with the reported numbers surely representing just the tip of the iceberg in light of the previously noted prevalence of physical and mental disability in prison (Prison Reform Trust, 2023) and the proportion of people in custody reporting difficulty undertaking daily activities of living (Tucker et al., 2019; Walton et al., 2023). The challenges faced in England are similar to those reported in Australia and the US, however there is a dearth of research globally specifically around social care on release from prison (Tucker et al. ,^2024^).

### Implications of findings

In our previous paper on the early arrangements in place for people released from prison we discussed the need for more robust screening and assessment processes, improved communication between authorities and the probation/social care interface (Robinson et al., 2021). In the remainder of this discussion, we now consider three further issues raised by the latest findings, namely the need for better data, the potential to make greater use of ROTL and the possible wider use of technology in planning the release of people to the community.

### The need for routine data collections

This study highlights the lack of data most local authorities have on the number and profile of people with social care needs released from prison. Yet such data is surely needed by policy makers, commissioners and providers at local and national level to enable individual authorities to effectively prepare people in prison with social care needs for their release to the community, inform the wider strategic geographical placement of social care and support services for people released from prison with social care needs and guide workforce planning (Her Majesty’s Inspectorate of Prisons & Care Quality Commission, 2018; Skills for Care, 2021). The introduction of systematic screening prior to people’s release and the development of local and national routine data collections to inform needs analysis and service planning at all levels of the criminal justice system is thus surely an imperative going forward. It is however, vital, that this is based on multiagency conversations about what it would be useful to collect for both individual local and national bodies – conversations which could also usefully explore what specific outcome measuring would be useful, with no such data currently reported.

### The potential utility of release on Temporary Licence

Whilst not all prisoners can apply for ROTL (exclusions include Category A prisoners and people on remand) our findings suggest that greater use could be made of ROTL in helping people with social care needs prepare for release, assessing their ability to live in the community and helping them acclimatise to life outside of prison, particularly people likely to have been institutionalised by a long stay in custody. Although the very low number of authorities who reported the regular use of ROTL may in part be related to the temporary suspension of ROTL during Covid, a previous study of the experience of a small sample of older people on release from prison also suggested ROTL was rarely used, but described it as having unique benefits for the resettlement of this group, enabling them to work or volunteer in the community and re-establish links with family (Cornish et al., 2016). Clearly there are risks with ROTL, including the risk that people will re-offend whilst out of prison on licence. However, the data suggest such risks are low and that its use is associated with reducing re-offending for those to whom it is given prior to release from prison (benefiting not only the individual, but the wider community too) and in 2019 the government published a new Policy Framework allowing prison governors more autonomy in releasing people on licence (Ministry of Justice, Her Majesty’s Prison Service and Her Majesty’s Prison and Probation Service, 2019; Nacro, 2023).

### The greater use of technology

Whilst technology is used in prison systems globally, its primary purpose is to collate electronic information and data and strengthen security and surveillance systems (Prais & Sheahan, 2019). The use of modern technology, however, also has the potential to facilitate the preparation of people in prison for release. Secure in-cell telephones have now been introduced in around two-thirds (64%) of prisons in England, enabling people to stay in touch with their families and loved ones (Prison Reform Trust, 2022), whilst some prisoners also have access to online video ‘visits’, although these are tightly regulated (Codd, 2020). Such technology could surely also be used to facilitate the undertaking of pre-release assessments by social workers in other authorities where distance or time preclude these happening in person, as well as having the potential to provide contact with the outside world, showing people where they will be living on release, for example. Indeed, in some high income countries virtual reality programming is now also helping people prepare for life beyond the prison walls, enabling people to experience everyday activities such as using a bank card or self-scan checkout (Prais & Sheahan, 2019) albeit there are of course issues around funding, infrastructure and staffing, as with all new technology.

### Methodological considerations

In assessing the findings from this study, a number of methodological considerations should be taken into account. First, the research only explored the local authorities’ perspective and lacks the voice of those individuals who require social care on release from prison. Second, although the 57 per cent response rate gives confidence in the representativeness of the findings, there was a higher response from authorities with prisons in their catchment area (68 per cent) who are likely to have seen the survey as more relevant to their concerns. Third, prisons are not homogeneous environments in terms of their physical environment, facilities or populations and some authorities will serve prisons with a higher number of people with social care needs than others and/or receive higher number of people with social care needs on release from prison, necessarily affecting the services required. Fourth, the survey was conducted in 2020 and focused on people’s experience in the previous 12 months. Whilst early respondents will have described their practice and experience pre the Covid pandemic, later authorities’ responses are more likely to have been influenced by the pandemic, which undoubtedly complicated release planning. There was, however, very little mention of Covid in the survey responses and government plans to release prisoners early did not materialise. Further, the issues the findings raise are arguably just as relevant now as during/before the pandemic.

## Conclusions

Whilst the failure to meet the social care needs of people released from prison has significant consequences for their day-to-day functioning, health and wellbeing, rehabilitation and likelihood of re-offending (Department of Health, 2014; Parker, McArthur and Poxton, 2007) the provision of social care for people released from custody has not generally been a priority for local authorities. This study found some signs that the Care Act and associated guidance have started to change this and suggests a number of ways in which local authorities are beginning to address this group’s needs. However, there are a lack of formal processes to address the social care needs of individuals being released from prison into the community. It also highlighted the urgent need for better data on the number and profile of this population and for research on the feasibility and effectiveness of different ways of delivering social care to improve social care outcomes. As such, perhaps it’s main contribution to the current evidence base is in identifying not only what we know, but what we don’t know and need to address next.

## Data Availability

The dataset analysed in the current study are not publicly available due to confidential participant information included in the interview transcripts.

## Abbreviations

ADASS: Association of Directors of Adult Social Services
LA: Local Authority
MAPPA: Multi-Agency Public Protection Arrangements
NIHR: National Institute for Health Research
ROTL: Release On Temporary Licence
